# Identification of a novel ALK intergenic fusion in a resected high‑recurrence‑risk lung adenocarcinoma and adjuvant treatment with ensartinib: a case report

**DOI:** 10.3389/fonc.2026.1826092

**Published:** 2026-04-29

**Authors:** Jing Li, Bo Ma, Ruiqing Mian, Yifan Wang, Lei Zhang, Na Yan, Jinxi He

**Affiliations:** 1 Department of General Thoracic Surgery, General Hospital of Ningxia Medical University, Yinchuan, China; 2 Key Laboratory of Digital Technology in Medical Diagnostics of Zhejiang Province, Dian Diagnostics Group Co. Ltd., Hangzhou, Zhejiang, China

**Keywords:** adjuvant therapy, ALK fusion, case report, ensartinib, intergenic region, lung adenocarcinoma

## Abstract

We report a novel intergenic anaplastic lymphoma kinase (ALK) fusion identified in a patient with resected stage IIIA lung adenocarcinoma. A 75-year-old woman underwent lobectomy for a left lower lobe tumor, with final pathology revealing pT3N2aM0 disease with high-risk features (pleural invasion, vascular tumor thrombus, and spread through air spaces). Comprehensive next-generation sequencing (NGS) of the surgical specimen identified a previously unreported in-frame fusion between an intergenic region of chromosome 6 (between STPLC1P2 and LOC100128365) and exon 20 of ALK on chromosome 2, retaining the entire kinase domain. Immunohistochemistry confirmed strong ALK protein expression (D5F3, Ventana). Based on the high-risk pathological stage and the presence of a targetable ALK driver, adjuvant therapy with the ALK tyrosine kinase inhibitor ensartinib was initiated instead of conventional chemotherapy. The patient tolerated treatment well, and a follow-up chest CT at approximately 6 months showed no evidence of local recurrence or distant metastasis. Long-term outcomes remain to be determined. This case expands the spectrum of ALK rearrangements and highlights the critical role of molecular profiling in guiding adjuvant treatment decisions for early-stage, high-risk non-small cell lung cancer (NSCLC).

## Introduction

Chromosomal rearrangements involving the anaplastic lymphoma kinase (ALK) gene are established oncogenic drivers in approximately 3-5% of non-small cell lung cancer (NSCLC). While the echinoderm microtubule-associated protein-like 4 (EML4)-ALK fusion is most common, next-generation sequencing (NGS) has unveiled a growing diversity of rare ALK fusion partners, including protein-coding genes, non-coding RNAs, and intergenic regions (1, 2). Several recent reports have demonstrated that intergenic region-ALK fusions, which preserve the ALK kinase domain, can be functional and confer sensitivity to ALK tyrosine kinase inhibitors (TKIs) in advanced NSCLC (3–5).

The management of completely resected ALK-positive NSCLC is evolving. The phase III ALINA trial recently established alectinib as a new standard of care for adjuvant therapy in stage IB-IIIA ALK-positive NSCLC, demonstrating a significant improvement in disease-free survival compared to chemotherapy (6). This underscores the importance of identifying actionable ALK fusions in the early-stage setting to optimize post-operative strategies.

In this report, we describe a case of a previously unreported ALK fusion present in the intergenic region between STPLC1P2 and LOC100128365, identified in a patient with resected stage IIIA lung adenocarcinoma. The fusion was associated with strong ALK protein expression. We herein present this case to describe the molecular finding and discuss the subsequent decision for adjuvant ALK-TKI therapy.

## Case presentation

**Figure 1:**
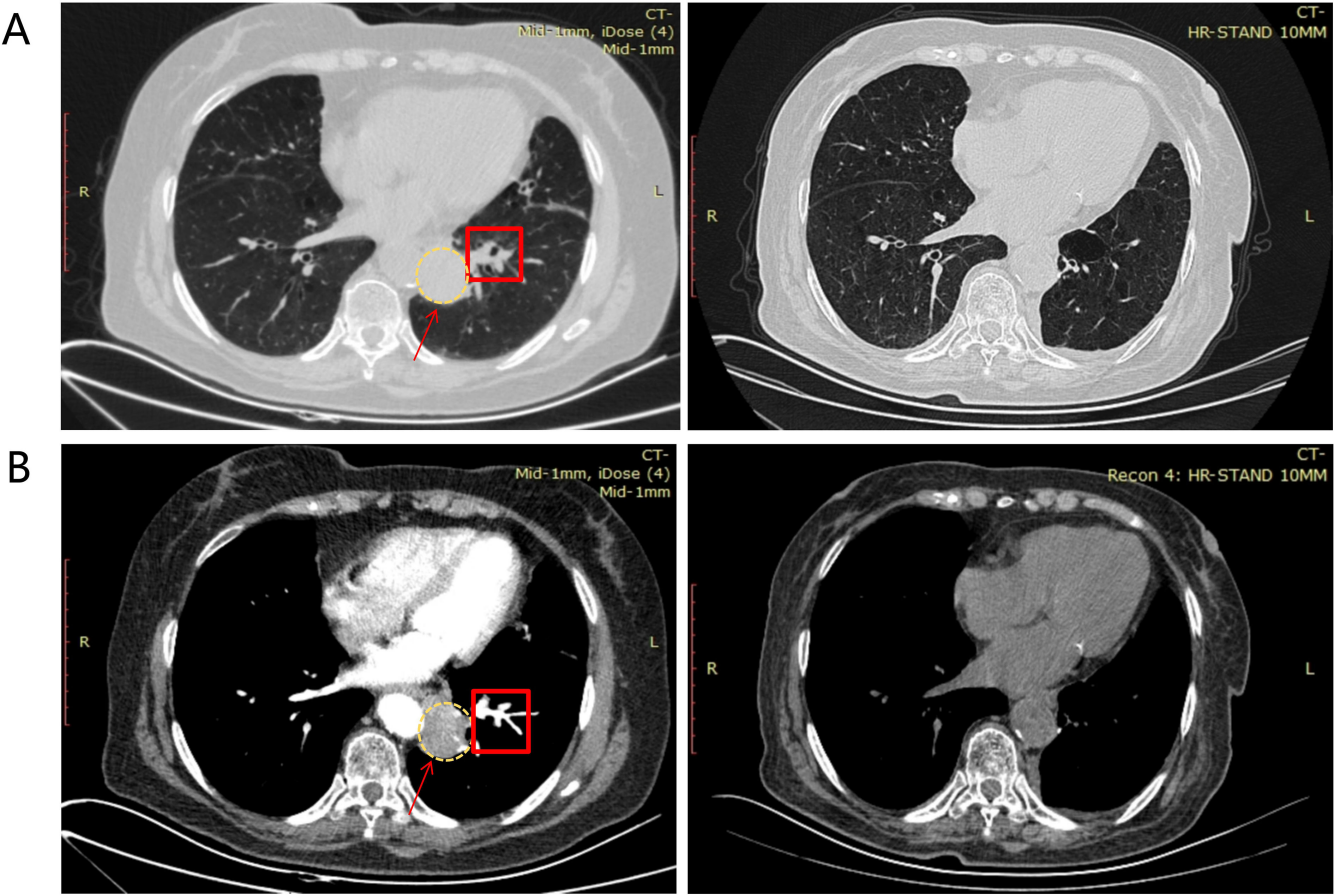
Radiographic evaluation of the patient. **(A)** Lung window and **(B)** mediastinal window images. For each panel, the left image is preoperative and the right image is approximately 6 months after initiation of adjuvant ensartinib. The primary lesion is indicated by a red arrow and a yellow dashed circle. Positive lymph node stations (preoperative) are marked by red squares. Postoperative changes following left lower lobectomy and lymph node dissection are observed on the right images, with no measurable residual tumor.

A 75-year-old non-smoking woman presented with a 4-month history of shortness of breath. A chest computed tomography (CT) scan revealed a 5.2 x 4.1 cm spiculated mass in the left lower lobe (**Figure 1A**). No significant hilar or mediastinal lymphadenopathy was reported on the preoperative enhanced CT. Abdominal contrast-enhanced CT, cranial contrast-enhanced MRI, and whole-body SPECT showed no distant metastatic lesions. A CT-guided percutaneous core needle biopsy of the mass was performed. Histopathological examination confirmed invasive adenocarcinoma. Based on this imaging and biopsy result, the patient was clinically staged as cT3N0M0 (stage IIB). It should be noted that invasive mediastinal staging was not performed at that time, which is a limitation of the preoperative evaluation and may have contributed to understaging.

**Figure 2:**
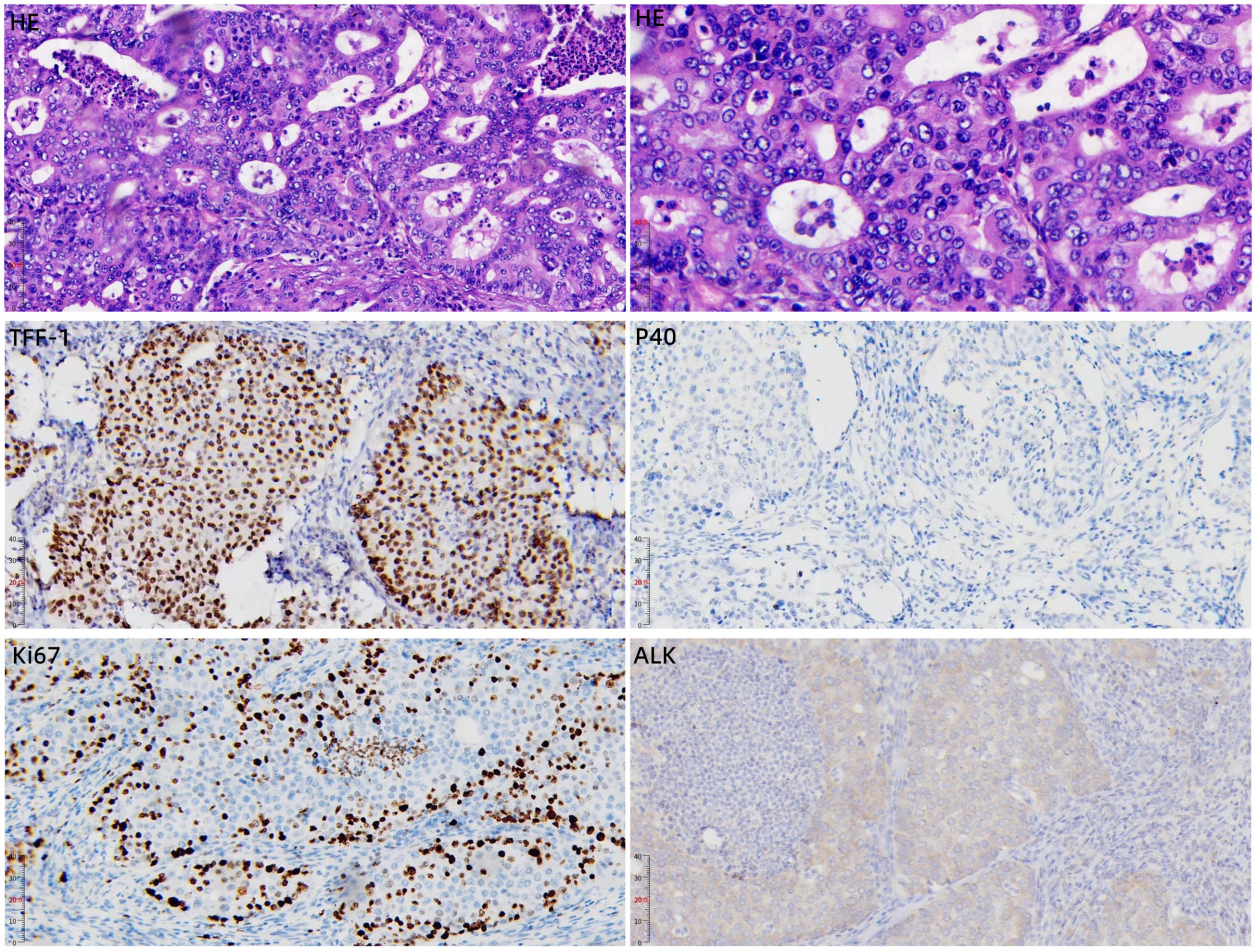
Representative histopathology and immunohistochemical staining of the resected lung adenocarcinoma specimen. The upper two panels show hematoxylin and eosin (H&E) staining at ×20 (left) and ×40 (right) magnification, confirming the solid subtype of invasive adenocarcinoma. The lower panels show immunohistochemical staining for TTF-1 (×20), p40 (×20), Ki-67 (×20), and ALK (×20, D5F3 antibody, Ventana). The tumor cells are TTF-1 positive and p40 negative, consistent with primary lung adenocarcinoma. Ki-67 labeling index is approximately 60%, indicating high proliferative activity. ALK shows diffuse cytoplasmic positivity, supporting the functional relevance of the detected intergenic region (between STPLC1P2 and LOC100128365)-ALK fusion.

The patient underwent a left lower lobectomy with systematic mediastinal lymph node dissection on September 15, 2025. The final pathological examination of the resected specimen, reported on September 19, 2025, revealed a 5.0 cm invasive adenocarcinoma of solid subtype (**Figure 2**). Immunohistochemical results showed: TTF-1 (+), P40 (-), Ki67 (60%) (**Figure 2**). The primary tumor invaded the visceral pleura (PL1) and exhibited vascular invasion (V1) as well as spread through air spaces (STAS). Critically, histological examination of the dissected lymph nodes confirmed metastatic carcinoma in the subcarinal (station 7, 1/1 node), hilar (station 10, 1/2 nodes), and interlobar (station 11, 1/4 nodes) stations, indicating the presence of occult lymph node metastases (pN2a) that were not evident on preoperative imaging. Consequently, according to the AJCC 9th edition, the final pathological stage was pT3N2a (stage IIIA), representing an upstaging from the preoperative clinical stage of cT3N0M0 (stage IIB).

**Figure 3:**
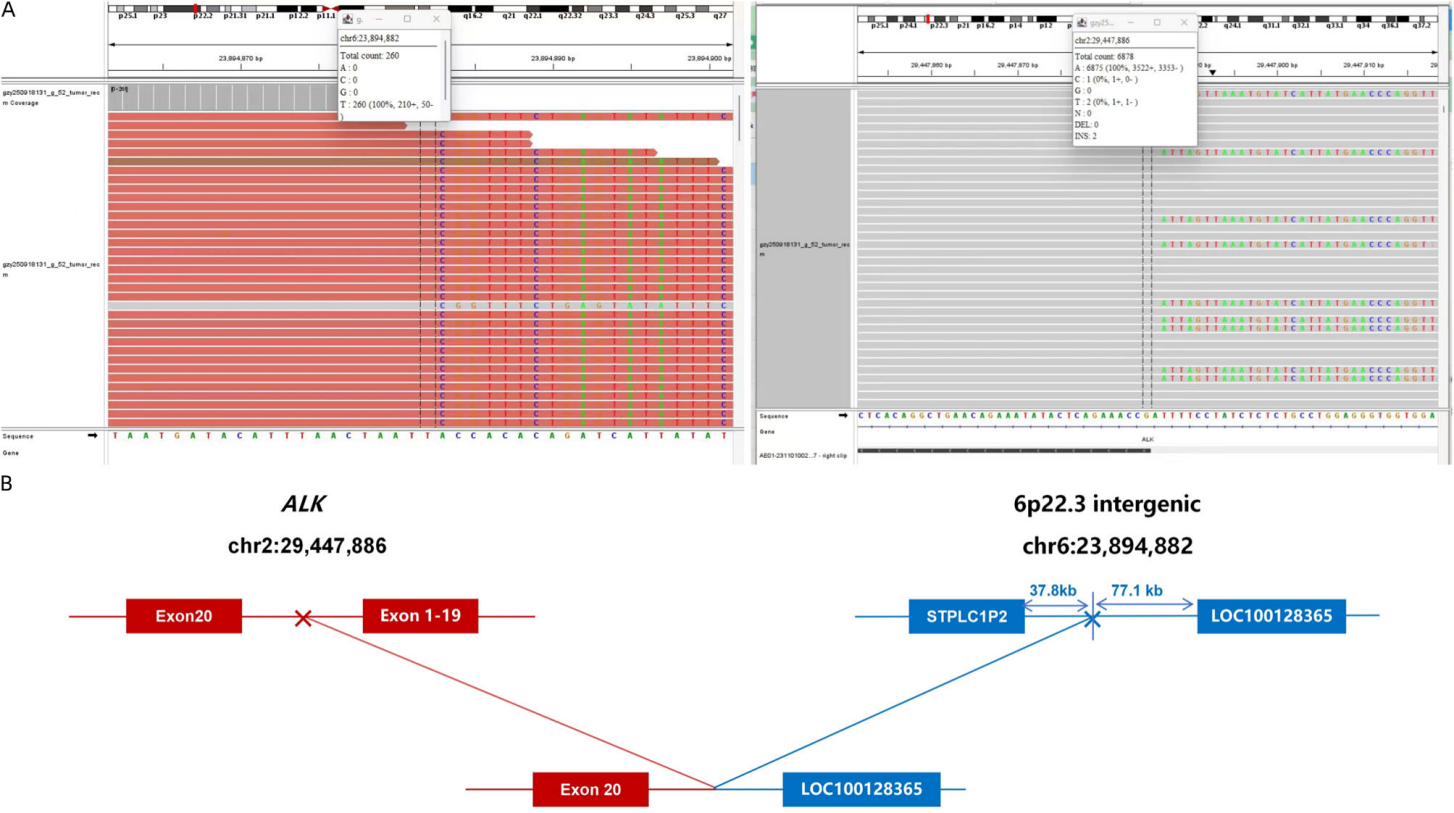
Molecular detection results of ALK fusion. **(A)** Integrated genomics viewer displaying the intergenic region (between STPLC1P2 and LOC100128365)-ALK fusion; **(B)** Schematic structure of the intergenic region (between STPLC1P2 and LOC100128365)-ALK fusion.

Given the high-risk pathological features (stage IIIA, PL1, V1, STAS), the tumor was submitted for comprehensive molecular profiling. Immunohistochemistry using the D5F3 antibody (Ventana) showed diffuse cytoplasmic ALK expression in tumor cells (**Figure 2**). Sequencing and analysis of excised tumor samples were performed using the Illumina NextSeq 500 platform and 52 gene plates related to targeted therapy and chemotherapy for lung cancer. The average sequencing depth is greater than 1000X, and the number of tumor cells in the sequencing sample is about 60%. Targeted DNA-based next-generation sequencing (NGS) identified a novel interchromosomal rearrangement, t(2;6). The breakpoints were located within an intergenic region on chromosome 6 (between the pseudogene STPLC1P2 and the long non-coding RNA gene LOC100128365) and within intron 19 of ALK on chromosome 2 (Mutation abundance 7.03%) (**Figure 3A**). The predicted fusion transcript retains exons 20–29 of ALK, which encode the entire intracellular tyrosine kinase domain, suggesting it may function as an oncogenic driver. (**Figure 3B**). No other canonical oncogenic drivers were detected.

The consensus highlighted the patient’s high risk of recurrence based on the postoperative upstaging to pT3N2a with adverse pathological features. The concurrent discovery of a strong ALK driver signal (IHC positive, NGS-confirmed novel fusion) provided a clear rationale for targeted therapy over conventional chemotherapy alone. Supported by evidence for TKI efficacy against similar intergenic fusions (3–5) and the ALINA trial paradigm, adjuvant ALK inhibition was recommended (6). The patient commenced oral ensartinib at a dose of 200 mg once daily on October 24, 2025. As of the last follow-up in April 2026, she tolerated the therapy well with no grade ≥2 adverse events. A follow-up chest CT scan showed no evidence of local recurrence or distant metastasis (**Figure 1B**). Long-term outcomes require continued follow-up.

## Discussion

This report describes a novel intergenic ALK fusion partner in a resected lung adenocarcinoma. The fusion involves an intergenic region between STPLC1P2 and LOC100128365 on chromosome 6. The fusion of the ALK gene retained the kinase domain, while IHC showed positive expression of ALK protein, indicating that this fusion is likely carcinogenic and may be sensitive to ALK inhibitors. The clinical significance of detecting such a rare fusion lies in its implications for treatment. In the metastatic lesions, multiple case reports have confirmed that intergenic region-ALK fusions are sensitive to various ALK-TKIs, including crizotinib, alectinib, and ensartinib (3–5, 7–9). The consistent theme across these reports is the preservation of the ALK kinase domain, which our case shares, and durable responses have been observed regardless of the specific intergenic partner (3, 5). Therefore, although functional studies were not performed, the structural prediction and IHC data provide a supportive, albeit indirect, evidence for targetability. base for targetability.

Our case uniquely situates this molecular finding in the context of adjuvant therapy for early-stage disease. The positive results of the ALINA trial have cemented the role of ALK-TKIs in the post-operative management of stage IB-IIIA ALK-positive NSCLC (6). Our patient’s pathological features (pT3N2a, V1, PL1, STAS) place her at the highest risk within this spectrum, justifying an aggressive adjuvant approach. The decision to use ensartinib rather than alectinib or platinum-based chemotherapy was made after multidisciplinary discussion and shared decision-making with the patient. Several factors informed this choice. First, at the time of treatment initiation, alectinib was not yet approved or reimbursed for adjuvant use in our region, limiting its availability for this patient. Second, the patient was 75 years old with no significant comorbidities; ensartinib has demonstrated a favorable tolerability profile in clinical trials and real-world studies, which was an important consideration for adjuvant therapy in an elderly patient. Third, although ensartinib has not been studied in the adjuvant setting, multiple published case reports have demonstrated efficacy of ALK inhibitors, including ensartinib, against intergenic ALK fusions structurally similar to the one identified in this patient (3–5). This provided indirect but clinically relevant support for the treatment decision. Therefore, ensartinib was selected as a non-standard, exploratory, individualized adjuvant treatment rather than as a substitute for established standards. We emphasize that this decision does not imply equivalence between ensartinib and alectinib in the adjuvant setting, and the choice should be interpreted within the specific clinical context of this case. Our case illustrates a scenario where individualized factors, particularly drug availability and patient tolerability considerations, led to a different treatment choice.

This case highlights several important points for clinical practice. First, it emphasizes the value of broad molecular profiling, including DNA-based NGS capable of detecting intergenic rearrangements, even in resected early-stage lung cancer, particularly for high-risk cases. Second, it illustrates a practical framework for clinical decision-making when a rare, potentially actionable variant is identified: integrating the biological plausibility of the variant (kinase domain retention, IHC positivity), cross-referencing published precedent for structurally similar fusions, and weighing the patient’s individual recurrence risk and fitness for various therapies.

The primary limitation of this report is the relatively short follow-up duration (approximately 6 months), which precludes meaningful assessment of long-term efficacy or recurrence prevention in the adjuvant setting. While the patient has shown no radiographic evidence of recurrence at nearly 6 months, this observation should not be interpreted as evidence of durable benefit. Continued long-term follow-up is essential to determine the ultimate clinical outcome. In addition, RNA−based confirmation of the fusion transcript was not performed. Although DNA−based NGS with split−read evidence strongly supports the presence of the rearrangement, the lack of RNA−level validation is a limitation, particularly for intergenic fusions where transcriptional outcomes may vary. Direct functional validation (e.g., *in vitro* kinase assays or cell−based transformation studies) was not carried out. While the retention of the ALK kinase domain and the concordant ALK protein expression by IHC provide supportive evidence for oncogenic potential, the absence of direct functional characterization remains a limitation. Therefore, the biological interpretation of this fusion remains inferential. Nonetheless, the structural and immunohistochemical data, together with published reports of structurally similar intergenic ALK fusions responding to ALK inhibitors, provided a basis for therapeutic exploration in this individual context.

## Conclusion

We identified a novel ALK intergenic fusion in a patient with high-risk, resected stage IIIA lung adenocarcinoma. Based on the retention of kinase domains and positive expression of ALK protein, it is speculated that this fusion may have carcinogenicity and potential targeting. This finding led to the initiation of adjuvant ensartinib in this individual case, illustrating how comprehensive molecular profiling can inform personalized treatment decisions in the adjuvant setting. The short-term tolerability was favorable, but long-term efficacy and recurrence prevention remain unknown, and continued follow-up is required. This case expands the known heterogeneity of ALK rearrangements and supports the growing rationale for targeted therapy in the adjuvant setting for genomically selected patients.

## Data Availability

The original contributions presented in the study are included in the article/supplementary material, further inquiries can be directed to the corresponding author/s.
